# Association of Perception of Front-of-Pack Labels with Dietary, Lifestyle and Health Characteristics

**DOI:** 10.1371/journal.pone.0090971

**Published:** 2014-03-12

**Authors:** Caroline Méjean, Pauline Macouillard, Sandrine Péneau, Camille Lassale, Serge Hercberg, Katia Castetbon

**Affiliations:** 1 Université Paris 13, Sorbonne Paris Cité, Equipe de Recherche en Epidémiologie Nutritionnelle (EREN), Centre de Recherche en Epidémiologie et Biostatistiques, Inserm U1153, Inra U1125, Cnam, Université Paris 5, Université Paris 7, Bobigny, France; 2 Unité de surveillance et d’épidémiologie nutritionnelle (USEN), Université Paris 13, Sorbonne Paris Cité, Centre de Recherche en Epidémiologie et Biostatistiques, Bobigny, France; 3 Department of Public Health, Hôpital Avicenne, Bobigny, France; 4 Institut de veille sanitaire (InVS), Département maladies chroniques et traumatisme, Unité de surveillance et d’épidémiologie nutritionnelle (USEN), Saint-Maurice, France; National Institute of Agronomic Research, France

## Abstract

**Objective:**

To identify patterns of perception of front-of-pack (FOP) nutrition labels and determine dietary, lifestyle and health profiles related to such patterns.

**Design:**

Cross-sectional.

**Participants/Setting:**

28,952 French adults participating in the web-based Nutrinet-Santé cohort.

**Outcome measures:**

Perception was measured using indicators of understanding and acceptability for three simple FOP labels (“green tick”, the logo of the French Nutrition and Health Program and “simple traffic lights” (STL)), and two detailed FOP formats (“multiple traffic lights” (MTL) and “color range” logo (CR)), placed on ready-to-eat soup packages. Dietary intake data were collected using three web-based 24 h records.

**Statistical analyses:**

Associations of perception patterns with individual characteristics, including diet, lifestyle and health status, were examined using analysis of covariance and logistic regression, adjusted for socio-demographic and economic factors.

**Results:**

No clear trend emerged concerning differences in dietary intake between perception groups. Low physical activity and obesity were more frequent in the ‘favorable to STL’ group (respectively, 20.7% and 10.7%). The ‘favorable to MTL’ group included the highest percentage of individuals who declared type 2 diabetes (2.2%). Persons with hypertension were proportionally more numerous in the ‘favorable to MTL’ and the ‘favorable to CR logo’ groups (respectively, 9.5% and 9.3%).

**Conclusions:**

After adjustment for socio-demographic and economic factors, no FOP label stood out as being more suitable than another for reaching populations with poor diet. However, both STL and MTL may be most appropriate for increasing awareness of healthy eating among groups at higher risk of nutrition-related chronic diseases.

## Introduction

Nutrition labeling on food packaging would appear to be of value in promoting healthy eating by helping consumers make appropriate food-buying decisions [1]. In recent years, new types of food labeling on the front of packages have been designed, providing simplified information on nutritional content at a glance, along with back-of-pack detailed energy and nutrient content information [1], [2]. Various front-of-pack (FOP) nutrition labeling formats now exist. The presence of FOP labeling is not mandatory on commercialized foods and in both the US and Europe, debate continues as to how such information should be provided [3]–[5]. In their review, Cowburn and Stockley have found that nutrition labels are not used by consumers because of the lack of understanding of terms and the concerns about the accuracy of the information [1]. Focusing on the acceptability and comprehension of FOP labels is therefore a valid step toward understanding their effect on consumer purchasing. Literature on factors which might affect their use is abundant [4]. Previous works showed that FOP labels differed in terms of market penetration, consumer acceptability and understanding [2], [4]–[13]. Most FOP labels are clearly understood [6], [7], [9], but compared to simple formats such as the ‘Green Keyhole’ and the ‘Pick the Tick’ [14], [15], detailed FOP labels (i.e. multiple traffic lights, MTL) appear to be more readily accepted by consumers in the general population, probably due to more rapid and easier identification and use, along with more complete information [2], [4], [8], [11], [16]–[19]. However, elucidation of the most appropriate FOP label formats for increasing awareness of healthy choices in groups at high risk of nutrition-related chronic diseases is needed. The study of variations in labeling perception according to socio-economic status, dietary pattern and obesity status might contribute to our information on this subject [4], [16].

Most previous studies comparing FOP label formats by specific target groups showed greater liking and better understanding of the traffic light (TL) system in the lowest socio-economic groups [2], [4], [6], [8], [17], [18], [20]. To our knowledge, few studies have focused on perception of FOP labels according to other individual factors such as BMI and perceived health status [6], [20]–[24]. In addition, no study has evaluated the association of FOP label perception with usual dietary intake or with intermediate risk factors for chronic diseases such as hypertension, type-2 diabetes and hypercholesterolemia. Moreover, differences in acceptability may be related to what the buyer expects from a label: a) reliability, easy identification, understanding and use; b) full information; or c) freedom of choice [2], [7]. However, the available studies did not take into account various dimensions constituting “acceptability” that would help to accurately define profiles of label perception and elucidate their relationship with individual factors.

Using a multidimensional approach, we had previously identified patterns of perception of FOP labels by clustering subjects into groups according to understanding and various aspects of acceptability [18]. The aim of the present epidemiological study was to determine whether dietary patterns, lifestyle and health characteristics are associated with cluster membership.

## Methods

### Study Population

Subjects were participants in the NutriNet-Santé Study, a large web-based prospective observational cohort launched in France in May 2009 with a scheduled follow-up of 10 years (recruitment planned over a 5-year period). It was implemented in a general population targeting internet-using adult volunteers aged 18 years or more. The study was designed to investigate the relationship between nutrition and health, as well as determinants of dietary behavior and nutritional status. The design, methods and rationale have been described elsewhere [18]. Briefly, participants had to fill in an initial set of questionnaires assessing dietary intake, physical activity, anthropometry, lifestyle and socio-economic conditions along with health status in order to be included in the cohort. Moreover, each month they are invited to fill in complementary questionnaires. In September 2009, 50,794 participants already included in the cohort were invited to complete an original web-based questionnaire on the Nutrinet-Santé website regarding acceptability and comprehension of FOP labels. Thus, these data were collected 1 to 4 months after collection of dietary, health and socio-demographic data.

### Ethics Statement

This study was conducted according to guidelines laid down in the Declaration of Helsinki, and all procedures were approved by the International Research Board of the French Institute for Health and Medical Research (IRB Inserm n° 0000388FWA00005831) and the Commission Nationale Informatique et Libertés (CNIL n° 908450 and n° 909216). Written electronic informed consent to participate in the study was obtained from all subjects.

### FOP Labels

#### Description of formats

**Figure 1:**
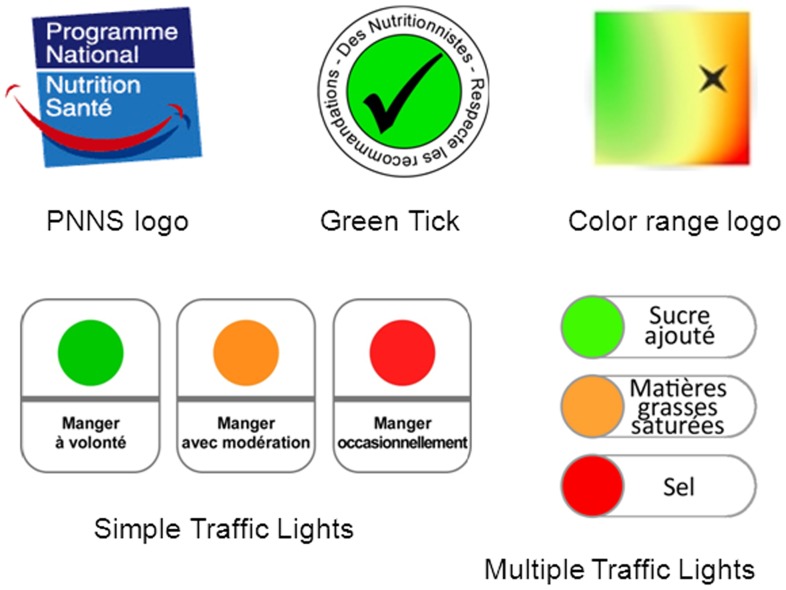
Front-of pack nutrition labels studied. Translation of French words: “Programme national Nutrition Santé”: National Nutrition and Health Program; “Respecte les recommandations des nutritionnistes”: Follows nutritionist recommendations; “Manger à volonté”: Eat plenty; “Manger avec modération”: Eat in moderation; “Manger occasionnellement”: Eat sparingly; “Sucre ajouté”: Added sugar; “Matières grasses saturées”: Saturated fat; “Sel”: Salt.

Five FOP labels placed on packages of ready-to-eat soups were tested (Figure 1 and Figure S1). The logo of the French Nutrition and Health Program (PNNS) has been widely used for media campaigns launched by the French Ministry of Health [25] and is therefore fairly familiar to the general population. The green tick, specifically designed for this study, was inspired by the ‘green keyhole’ symbol developed by the Swedish Food Administration [14], and by the ‘Pick the Tick’ program introduced by the Heart Foundation in Australia and New Zealand [15]. The PNNS logo and the green tick simply indicate an overall positive evaluation of a product. The “simple traffic lights” logo (STL) developed in the UK by the Food Standards Agency proposes either a positive, neutral or negative judgment, plus advice relating to consumption frequency [17]. The “color range” or CR logo was specifically designed for our study (Figure 1). It enables positioning products on a color scale of nutritional quality for food products and thus does not provide a categorical judgment. It uses a continuous color gradation from green to red, passing through orange and yellow intermediate areas. The MTL symbol, also recommended by the UK Food Standards Agency [17], provides a separate evaluation of several nutrients (positive, neutral or negative) and, in our case, of added sugar, saturated fat and sodium.

#### Achievement criteria for labels

We used the “SAIN LIM” system [26] that classifies foods into four nutrient categories based on the nutrient content of each soup product, so that use of the tested logos could be defined (Figure S1). Vegetable soup had the most favorable nutrient profile (class 1), while boiled chicken soup characterized foods with the least favorable nutrient profile (class 4). Chinese soup corresponded to class 3, intermediate in terms of nutritional quality (high nutrient density, high content of unfavorable nutrients).

In the introduction to the questionnaire, we briefly explained the various label formats tested and their theoretical use. Participants were informed that the PNNS logo and green tick were given only to the healthiest foods (class 1), and the PNNS logo was certified by the French Ministry of Health, whereas the green tick meant that nutritionists guaranteed the nutritional quality of foods. STL consisted of either a green (“eat plenty”), amber (“eat in moderation”) or red (“eat sparingly”) symbol depending on how food was classified: classes 1, 3 and 4, respectively. The CR logo was one hypothetical representation of the SAIN LIM system for classifying and positioning foods on two continuous scales for “positive” and “unfavorable” nutrient contents [26]. We explained to participants that the closer the star came to the green area (upper left), the healthier the nutrient profile of the product (class 1). Conversely, the closer the star came to the red area (bottom right), the less healthy the nutrient profile of the product (class 4). An example was given (Figure 1). For MTL, green, amber and red indicated low, moderate and high contents, respectively, of added sugar, saturated fat and sodium based on thresholds defined by the SAIN LIM system for these unfavorable nutrients [26].

### Data Collection

#### Comprehension and acceptability of FOP labels

The specific web-based questionnaire evaluated strengths and weaknesses of each FOP label in terms of comprehension and acceptability. Acceptability was based on several criteria: attitude, liking, visual attractiveness and perceived cognitive workload needed for interpreting it. These dimensions were inspired by the framework of system acceptability developed by Nielsen and used in information and communication technology [27]. A within-subjects design was used and all questions concerning acceptability and understanding were repeated for each logo and for each participant. The order of presentation of the label formats was as follows: first, the Green Tick, then STL, the CR logo, the PNNS logo and finally MTL. Participants would not be able to find any logical reason for this order.

To assess comprehension, participants were asked to evaluate the “healthiness” of each given product. They were shown photographs of the three soup variants described above, unbranded, with no information on nutrition facts (Figure S1). For each label, participants had to determine, among three corresponding photographs, whether five statements were true or false or whether the label did not enable a judgment. Statements were as follows: “it is inadvisable to eat boiled chicken soup” (correct answer: false), “Chinese soup is less salty than boiled chicken soup” (correct answer: “the label does not enable me to answer”, except for the MTL, for which the correct answer was “false”), “vegetable soup has a good nutritional profile” (correct answer: “true”), “vegetable soup is the one that contains the least salt” (correct answer: “the label does not enable me to answer”, except for the MTL, for which the correct answer was “true”) and “only vegetable soup should be eaten” (correct answer: false). A comprehension score was calculated and classified into three levels: high (four or five correct answers), medium (three correct answers) and low (two, one or no correct answers).

For each FOP label, participants chose the statement best reflecting their attitude toward that label, i.e. their intent to use the label. Rejection was reflected in the sentence ‘I am not influenced by this label’. Participants were considered confused if they chose the statement ‘I no longer know what to choose’. The statement ‘This label is helpful, but I would rather form my own opinion on nutritional facts’ indicated lack of interest. Acceptance was measured by statements such as: ‘I would choose certain food products based on this label’. Finally, the statement ‘I would choose all my food products based on this label’ reflected total acceptance.

Liking was evaluated by the question ‘Would you like to see this FOP label on food products?’ for each label. Moreover, at the end of the questionnaire, FOP label formats were compared for liking by asking participants to choose their preferred label, the one they least appreciated, the one that most influenced them, the one they wanted to see on the front of packages and the one that best helped them choose healthy products.

Attractiveness of the label format grouped together measurements of perception of the potential qualities of a format. It was evaluated by its perceived contribution to information, ease of identification and reliability. Perceived contribution to information was measured by statements such as “this FOP label provides me with the information I need”, “this FOP label gives too much information” or “this FOP label does not give enough information”. Ease of identification was evaluated by the statement “this FOP label is easy to identify”. Finally, reliability was measured by the statement “this FOP label provides reliable information”. These indicators were assessed by individual questions on a 5-point rating scale, from ‘fully agree’ to ‘fully disagree’. They were classified into three categories according to data distribution. At the end of the questionnaire, we asked participants to select the most informative and reliable label.

Perceived cognitive workload grouped together a set of measurements of perception of potential format defects. It was assessed by 3 indicators: complexity of understanding, time span needed for interpreting the logo and discomfort occasioned by the message. The complexity level of understanding was evaluated by the following statements: “this FOP label is too complex for understanding” and “this FOP label is easy to understand”. Perceived time span needed for interpretation was measured by the statements “this FOP label takes too long to understand” and “this FOP label permits rapid understanding of the information”. Finally, perceived discomfort was measured by the statement “this FOP label makes me uncomfortable”. These were measured using 5-point rating scale questions, from “fully agree” to “fully disagree”. These three indicators were classified into three categories according to data distribution. At the end of the questionnaire, FOP label formats were compared for perceived cognitive workload. We asked participants to choose, among the five formats, the most complicated label, the one requiring the longest time span for interpretation and the label that made them feel most uncomfortable.

#### Assessment of individual characteristics

Dietary intake, health and socio-demographic data were collected at enrollment in the cohort. Dietary data were collected using three web-based 24 h dietary records randomly assigned over a two-week period (1 weekend day and 2 weekdays). The dietary record relies on a meal-based approach, recording all foods and beverages (type and quantity) consumed at all eating occasions [28]. First, the participant fills in the names of all food items eaten. Next, the participant estimates portion sizes for each reported food and beverage according to standard measurements or using photographs available via the interactive interface, taken from a validated picture booklet [29]. Consumption of fish and seafood per week was assessed by a specific frequency question. The nutritional values for energy, macronutrients and main micronutrients were estimated using published nutrient databases [30], [31] and completed for recent market foods and recipes.

Physical activity was assessed using the short form of the International Physical Activity Questionnaire, in the French language, validated against doubly labeled water [32], [33]. BMI was assessed using self-reported height and weight. Status with respect to type-2 diabetes, hypertension and hypercholesterolemia was given by participants when answering questions in the questionnaire on specific health items and current use of medication. Socio-demographic and economic factors included gender, age, marital status, having at least one child or not, educational level and occupational category.

### Statistical Analysis

The present analyses focused on participants included in the Nutrinet-Santé cohort study who had completed the questionnaire measuring perception of FOP labels and at least three 24 h dietary records and who had no missing socio-demographic, anthropometric or health data. For each participant, daily mean quantities of food groups (in grams) and nutrient intake were calculated from the 24 h records, weighted according to the day (week or weekend). Diet-underreporting subjects were identified by the method proposed by Black [34]. Briefly, basal metabolic rate (BMR) was estimated by Schofield equations [35] according to sex, age and weight and height collected at enrolment in the study. BMR was compared to energy intake taking into account the physical activity level. A physical activity level of 0.88 was used to identify extremely underreporting subjects, and a physical activity level of 1.55 was used to identify other underreporting participants [34]. Within 24 h records, the participant declared whether the reported consumptions were fairly representative of his/her usual diet or strongly differed due to specific events (illness, a social event, etc.). These comments, such as acute disease and information collected at enrolment regarding current restrictive diet or a recent loss of weight (≥5 kg) were investigated to identify specific conditions that could objectively explain low energy intake. Participants who provided such information were not considered to be underreporters, whereas other underreporting participants were excluded from the analysis. In addition, erroneous quantities due to data entry errors were identified using day- and food-specific established thresholds. According to the percentage of erroneous data in declared quantities in the record and the declaration of the subject the representativeness of the record compared to his/her usual diet, the record was deleted or corrected.

Recommendations for food group servings were defined using the French recommendations of the PNNS [36]. Adherence to PNNS recommendations was defined for each food group as follows: ≥3 servings per day <6 for ‘grains’; ≥5 servings per day for ‘fruits and vegetables’; (≥1 servings per day <2 for ‘meat, seafood and eggs’; ≥2 servings per week for seafood; and ≥2.5 servings per day <3.5 for ‘dairy products’ for persons under 55 years of age and ≥2.5 servings per day <4.5 for older individuals. Thus, percentages of subjects who met recommendations were evaluated. In addition, we calculated the PNNS Guideline Score (PNNS-GS), a dietary quality score based on adherence to these recommendations [36]. It included 13 components, including foods (fruits and vegetables, grains, whole-grain food, dairy products, meat, seafood and eggs, only seafood, added fats, types of added fat, sweetened foods), beverages (alcoholic and non-alcoholic beverages), salt, and physical activity. The general approach for scoring was to attribute points according to how participants met with recommendations. Raw scores ranged from 1 (due to a negative half point for sweetened foods and salt) to 15. Penalties were applied to participants who had an energy intake that was 5% higher than their needs, as estimated using the basal metabolic rate and physical activity level. In a French study, representative of the general population, the mean PNNS-GS was 7.67±0.17 (range 1.2 to 15.0) in men and 8.55±0.12 (2.2 to 14.0) in women [37]. Data obtained using the IPAQ were computed for the metabolic equivalent task in min per week following recommendations of the IPAQ group (available at http://www.ipaq.ki.se/scoring.pdf). Recommended categories of physical activity (low, moderate, and high) were used in analyses. Normal weight, overweight and obesity were defined according to the WHO classification for BMI, as BMI <18.5, 25≤ BMI <30 kg/m^2^ and BMI ≥30 kg/m^2^, respectively [38].

Since “acceptability” comprised various complementary but overlapping dimensions, overall perception of FOP labels was not captured using traditional multivariate techniques. To identify patterns of perception of FOP labels by grouping subjects into distinct groups according to acceptability and understanding of FOP label formats, we used cluster analysis, for which the procedure has been described elsewhere [18]. Briefly, because of the large amount of data, the two-step cluster analysis procedure was based on the 58 variables relating to acceptability and understanding of each FOP label and comparisons between FOP labels [39], [40]. We used pseudo-F statistics for estimating the appropriate number of clusters [41]. For external validation and the description of perception patterns, characteristics of each cluster were described, comparing variables of acceptability and understanding of responders between clusters.

The relationship between cluster membership and dietary intake, lifestyle and health characteristics was examined using analysis of covariance for quantitative variables (nutrients intakes, PNNS-GS) and logistic regression for qualitative variables (meeting or not the nutritional recommendations, physical activity level, weight and health status). Relating to intake of nutrients and food groups, models were adjusted for energy intake and socio-demographic and economic characteristics. Models for physical activity and health characteristics were also adjusted for socio-demographic and economic factors.

Given the large size of our sample, significant differences between perception clusters were observed for most dietary, lifestyle and health factors. We therefore described only those results for which the difference in percentages and means between the cluster which presented the lowest value and the cluster with the highest value was >10%.

In order to correct the effect of under and over-representation of certain groups in the sample compared to the French general population, weighting, assigning a weight to each individual, was calculated separately for each gender according to national census data on age, educational diploma, area of residence and whether or not the household included at least one child, using the iterative proportional fitting procedure [42]. Two-sided tests and a P-value <0.05 were used for statistical significance. Data management and statistical analyses were performed using SAS (version 9.1; SAS Institute, Inc., Cary, NC, USA).

## Results

A total of 39,370 persons had completed the questionnaire regarding perception of FOP labels, i.e. 77.5% of volunteers included in the Nutrinet-Santé cohort at that time. We excluded 1,817 individuals who had not provided at least three 24 h dietary records during the first year of follow-up, 20 individuals with defective records, 751 participants with missing data for socio-demographic, weight, height and health status and 6,610 underreporting participants, thus leaving 28,952 participants available for analysis. No sociodemographic and economic difference was observed between our analysis sample and the entire sample (n = 38,619; that only excluded persons with sociodemographic missing data), except for the education level in the ‘favorable to STL’ group for which percentage of high educated persons was higher in the analysis sample. In addition, there is no difference between samples regarding variables assessing acceptability and understanding of FOP labels that constructed clusters.

### Description of Perception Patterns

As perception clusters were constructed from numerous variables, the description of clusters presents only variables assessing acceptability and understanding of FOP labels best enabling distinction between clusters (Table S1). Cluster analysis yielded four groups: 1) the ‘favorable to MTL’ group (68.5%); 2) the ‘favorable to green tick and PNNS logo’ group (20.5%); 3) the ‘favorable to STL’ group (10.3%); and 4) the ‘favorable to CR logo’ group (2.8%).

#### ‘Favorable to the MTL’ group

Cluster 1 (‘favorable to the MTL’ group) was shaped by a greater liking and a higher attractiveness for the MTL compared to the other labels (Table S1). It was also characterized by greater dislike of and a higher perceived cognitive workload involved in the CR logo. This cluster also showed better understanding of all labels.

#### ‘Favorable to green tick and PNNS logo’ group

Cluster 2 (‘favorable to green tick and PNNS logo’ group) was shaped by greater liking, higher attractiveness and a better understanding of the green tick and, secondarily, of the PNNS logo. It was also characterized by a high percentage of individuals who thought that no label should be present on the front of packages (20.9%) and that none provided necessary information (24.4%).

#### *‘*Favorable to the STL’ group

Cluster 3 (‘favorable to the STL’ group) was shaped by higher liking and greater attractiveness for STL and a higher perceived cognitive workload for the CR logo. Moreover, in this cluster, there existed a greater number of persons who better understood the simple logos, i.e. the green tick, STL and PNNS logos (Table S1).

#### ‘Favorable to the CR logo’ group

Cluster 4 (‘favorable to the CR logo’ group) was shaped by higher liking and greater attractiveness for the CR logo, with greater dislike concerning the PNNS logo. It was also characterized by a high percentage of individuals who felt that none of the labels tested, including the MTL, required a high cognitive workload. Finally, this cluster included numerous individuals who only poorly understood the MTL, whereas a higher level of understanding was found for the green tick and PNNS logos.

Socio-demographic and economic profiles of the perception clusters are presented in Table 1. All associations of socio-demographic characteristics with dietary, lifestyle and health factors were significant (p<0.0001) (data not shown). Unhealthier dietary profiles and higher prevalence of obesity, low physical activity, declared type-2 diabetes, hypercholesterolemia and hypertension were observed in men, single persons and those falling into low socio-economic categories (data not shown). Younger individuals presented unhealthier dietary intake, but a lower prevalence of intermediate risk factors for chronic diseases.

**Table 1:** Socio-demographic profiles of perception clusters, n = 28, 952 (Nutrinet-Santé Study, 2009–2010)^a^.

	Total samplen = 28 952(% or mean (sd))		“Favorable to MTL”group^b^ n = 19 842(% or mean (sd))	“Favorable to green tickand PNNS logo” groupn = 5 932 (% or mean (sd))	“Favorable to STL”group^d^ n = 2 973(% or mean (sd))	“Favorable to CRlogo” group^e^ n = 808(% or mean (sd))
**Gender**						
Men	47.47		47.48	48.16	42.09	61.59
Women	52.53		52.52	51.84	57.91	38.41
**Age, years**	47.07 (14.30)		45.34 (13.80)	52.61 (14.80)	41.92 (13.90)	53.44 (15.56)
**Marital status**						
Married/living with a partner	73.63		73.83	74.62	71.99	68.57
Divorced/separated/widowed/single	26.37		26.17	25.38	28.01	31.43
**At least one child at home**						
Yes	35.32		39.02	27.06	37.26	23.11
No	64.78		60.98	72.94	62.74	76.89
**Education level**						
Elementary school	4.90		4.11	6.32	5.25	9.02
Secondary and high school	44.14		41.95	49.51	48.58	40.80
College graduate	28.65		29.80	25.00	30.04	26.73
Advanced degree	22.31		24.14	19.17	10.31	23.44
**Occupational category**						
Self-employed, farmer	6.35		6.13	6.06	5.38	13.17
Managerial staff	23.60		23.25	26.33	15.55	28.90
Intermediate profession (skilledworkers or employees, primaryschool teacher, nurse, …)	24.87		25.35	24.24	21.80	25.94
Employee, manual worker	41.17		40.91	40.52	52.05	29.12
Never-employed	4.01		4.36	2.85	5.22	2.87

a All p-values were <0.0001.

b MTL, multiple traffic lights.

c PNNS, French Nutrition and Health Program.

d STL, simple traffic lights.

e CR, color range.

### Association of Perception Profiles with Dietary Intake, Lifestyle and Health Characteristics

Results of models before adjustment for socio-demographic and economic variables are presented in Tables S2, S3 and S4. After adjustment for socio-demographic and economic factors, no difference >10% between clusters was found for energy or nutrient intakes (Table 2). Mean (SE) PNNS guideline scores were, respectively, 9.18 (0.02) for the ‘favorable to MTL’, 9.06 (0.03) for the ‘favorable to green tick and PNNS logo’ and the ‘favorable to STL’ groups and 9.15 (0.05) for the ‘favorable to CR logo’ group. The observed difference between clusters was too small to be interpreted, even though a statistically significant result was found. The ‘favorable to CR logo’ group included the highest proportion of low consumers of “fruits and vegetables” and dairy products (Table 3). It also contained the highest proportion of participants showing overconsumption of dairy products, along with the ‘favorable to MTL’ group. In addition, the highest percentage of low consumers of “meat, seafood and eggs” was observed in the ‘favorable to CR logo’, the ‘favorable to MTL’ and the ‘favorable to green tick and PNNS logo’ groups. The latter group also included the highest percentage of participants with high alcohol consumption. Finally, low consumers of grains and seafood were more frequently present in the ‘favorable to STL’ group.

**Table 2:** Nutrient intake of perception clusters adjusted for energy intake and socio-demographic and economic factors, n = 28, 952 (Nutrinet-Santé Study, 2009–2010).

	Total samplen = 28 952		“Favorable toMTL” group an = 19 842		“Favorable to greentick and PNNS logo”group b n = 5 932		“Favorable toSTL” group cn = 2 973		“Favorable toCR logo”group d n = 808		
	Mean	SE	Mean	SE	Mean	SE	Mean	SE	Mean	SE	P-value
**Energy intake** e	2091.34	5.41	2093.42	4.92	2074.01	6.93	2088.87	9.22	2140.90	14.05	<0.0001
**Proteins** **(% of energy intake)** e	16.60	0.04	16.74	0.04	16.66	0.05	16.76	0.07	16.69	0.09	0.35
**Lipids** **(% of energy intake)** e	37.40	0.07	37.36	0.08	37.28	0.09	37.61	0.12	37.71	0.19	0.02
**Saturated fat** **(% of energy intake)** e	15.09	0.04	14.95	0.05	14.98	0.05	15.07	0.07	15.19	0.10	0.05
**Carbohydrates** **(% of energy intake)** e	41.94	0.08	41.76	0.09	41.87	0.11	41.63	0.15	41.52	0.22	0.25
**Simple carbohydrates** **(% of energy intake)** e	19.61	0.06	19.57	0.07	19.50	0.09	19.77	0.11	19.07	0.17	0.002
**Fibers (g/day)** f	19.19	0.08	19.22	0.07	19.23	0.11	19.00	0.13	18.80	0.20	0.06
**Sodium (mg/day)** f	2528.52	8.04	2538.40	7.44	2540.15	10.53	2476.03	12.89	2498.71	19.69	<0.0001
**Cholesterol (mg/day)** f	331.05	1.12	331.77	1.28	325.56	1.79	333.49	2.38	339.47	3.63	0.0001
**Iron (mg/day)** f	12.76	0.05	12.80	0.05	12.69	0.06	12.57	0.08	12.75	0.12	0.005
**Calcium (mg/day)** f	875.52	3.38	896.97	2.97	889.10	4.01	896.41	5.30	900.86	8.13	0.19

a MTL, multiple traffic lights.

b PNNS, French Nutrition and Health Program.

c STL, simple traffic lights.

d CR, color range.

e The model was adjusted for socio-demographic and economic factors, i.e. sex, age, marital status, at least one child at home, educational level and occupational category.

f The model was adjusted for energy intake and socio-demographic and economic factors, i.e. sex, age, marital status, at least one child at home, educational level and occupational category.

**Table 3:** Food group intakes of perception clusters adjusted for energy intake and socio-demographic and economic factors, n = 28, 952 (Nutrinet-Santé Study, 2009–2010)^a^.

	Total samplen = 28 952(%^f^)	“Favorable to MTL”group^b^ n = 19 842(%^f^)	“Favorable to greentick and PNNS logo”group^c^ n = 5 932 (%^f^)	“Favorable toSTL” group dn = 2 973 (%^f^)	“Favorable to CRlogo” group en = 808 (%^f^)
**Fruits and vegetables (servings/d)**					
Low consumption (≥0–<3.5 )	31.54	30.49	34.02	30.70	37.54
Moderate consumption (≥3.5–<5)	22.67	23.08	23.11	21.91	16.32
Adherence to recommendation (≥5)	45.79	46.43	42.87	47.39	46.14
**Grains (servings/d)**					
Low consumption (≥0–<3 )	54.31	54.12	52.74	55.59	51.94
Adherence to recommendation (≥3–<6)	41.74	41.61	42.81	42.03	41.74
High consumption (≥6)	3.95	4.27	4.45	2.38	6.32
**Milk and dairy products (servings/d)**					
Low consumption (≥0–<2.5)	52.83	52.22	53.99	53.43	55.27
Adherence to recommendation(<55 y: ≥2.5–<3.5; ≥55 y: ≥2.5–<4.5)	28.19	28.16	29.05	27.99	25.44
High consumption(<55 y: >3.5; ≥55 y: >4.5)	18.98	19.62	16.96	18.58	19.29
**Meat and poultry, seafood. and eggs (servings/d)**					
Low consumption (≥0–<1 )	27.31	27.58	27.78	24.62	27.34
Adherence to recommendation (≥1–<2)	55.38	54.97	54.93	58.78	56.06
High consumption (>2)	17.31	17.45	17.29	16.60	16.60
**Seafood (servings/wk)**					
Low consumption (<2)	54.69	53.69	55.65	58.55	56.00
Adherence to recommendation (≥2)	45.31	46.31	44.35	41.45	44.00
**Alcohol (ethanol g/d)**					
High consumption(>20 for women and >30 for men)	11.33	11.97	13.46	12.68	10.42
Moderate consumption(≤20 for women and ≤30 for men)	69.63	69.39	68.27	67.55	71.50
Abstainers and irregular consumers(< once a week)	19.04	18.66	18.30	19.75	18.14

a All p-values were <0.0001.

b MTL, multiple traffic lights.

c PNNS, French Nutrition and Health Program.

d STL, simple traffic lights.

e CR, color range.

f For each variable, percentages were adjusted for energy intake and socio-demographic and economic factors, i.e. sex, age, marital status, at least one child at home, educational level and occupational category.

The ‘favorable to STL’ group included the highest percentage of obese persons and those with low physical activity levels (Table 4). The ‘favorable to MTL’ group included the highest percentage of individuals with type 2 diabetes. In addition, the highest prevalence of hypertension was found in the ‘favorable to MTL’ and ‘favorable to CR logo’ groups. The ‘favorable to CR logo’ group included the highest proportion of persons with hypercholesterolemia.

**Table 4:** Lifestyle and health profiles of perception clusters adjusted for socio-demographic and economic factors, n = 28, 952 (Nutrinet-Santé Study, 2009–2010)^a^.

	Total samplen = 28 952(%^f^)	“Favorable toMTL” group^b^n = 19 842 (%^f^)	“Favorable to greentick and PNNS logo”group^c^ n = 5 932 (%^f^)	“Favorable toSTL” group^d^n = 2 973 (%^f^)	“Favorable toCR logo” group^e^n = 808 (%^f^)
**Physical activity level (min/d)**					
Low (<30)	18.79	18.59	18.68	20.73	17.89
Moderate (≥30–<60)	18.05	18.34	16.69	17.67	14.03
high (≥60)	63.16	63.07	64.63	61.60	68.08
**Body mass status (kg/m2)**					
Normal (<25)	66.09	65.93	67.09	65.55	65.30
Overweight (≥25–<30)	24.79	24.90	24.59	23.73	26.42
Obese (≥30)	9.12	9.17	8.32	10.72	8.28
**Self-reported** **type 2 diabetes**	1.94	2.24	1.17	1.94	1.03
**Self-reported** **hypertension**	9.06	9.52	7.69	8.67	9.33
**Self-reported** **hypercholesterolemia**	9.48	9.59	8.60	9.80	11.04

a All p-values were <0.0001, except for model regarding hypercholesterolemia adjusted for socio-demographic and economic factors for which the p-value = 0.04.

b MTL, multiple traffic lights.

c PNNS, French Nutrition and Health Program.

d STL, simple traffic lights.

e CR, color range.

f For each variable, percentages were adjusted for socio-demographic and economic factors, i.e., sex, age, marital status, at least one child at home, educational level and occupational category.

## Discussion

Relationships between acceptability, understanding and use of FOP labels and population characteristics have rarely been explored, particularly regarding persons with chronic diseases [2], [4]. The present epidemiological study, showing associations between FOP label perception and dietary, lifestyle and health profiles, emphasizes the importance of considering individual factors for developing effective formats to help consumers make healthier food choices. Effective nutrition labeling policies should be particularly attentive to the fact that the failure of FOP labels to achieve the reduction of diet- related diseases could be particularly marked in populations with higher nutritional risks, such as obese individuals [16]. For instance, those most concerned with managing their weight, although interested in food labels, are also most likely to underestimate the caloric content of meals, in both healthy and unhealthy products, that may result in weight gain [23]. Thus, our study provides useful information on the most appropriate formats for increasing awareness of healthy eating among targeted groups at higher risk of nutrition-related chronic diseases. Indeed, our study indicates a higher prevalence of intermediate risk factors for chronic disease in groups which presented better acceptability and understanding of the TL system (STL and MTL). In addition, our findings do not point out clear trends concerning differences in dietary patterns between perception groups. However, our results should be useful to public health authorities so as to improve specific food group consumption, by implementing FOP labels better accepted in populations which do not meet recommendations for these food groups.

The originality of our study lies in the comparison of different FOP labels via capture of multidimensional information on acceptability. The advantage of measuring all these aspects of acceptability is that they differ, but may overlap; consequently, they are complementary to each other, enabling better understanding of the acceptability mechanisms of label formats. As explained by Hersey et al., perception can lead to understanding that might direct the consumers’ decision making process and prompt the consumer to make healthier food purchases and, thus, healthier consumption choices [43]. Although our study cannot allow evaluating if some labels motivate participants to change behavior, our theoretical approach is complementary to experimental works that assessed the use of FOP labels and their impact upon purchasing behavior [6], [10], [23]. Indeed, our epidemiological study provides useful information, based on a large heterogeneous sample, regarding individual factors related to comprehension and acceptability of FOP labels, factors which might affect their use. In addition, the novelty in our study is to evaluate the associations between FOP labels perception and usual dietary intake, lifestyle or with intermediate risk factors for chronic diseases, which has never been studied.

Interpretation of the present results must take into account several limitations. Since the sample is not random, subjects are in better health and healthier lifestyle than the general population with lower prevalence of hypertension, type-2 diabetes, hypercholesterolemia and obesity, high physical activity level and higher adherence to the recommendations for fruit and vegetables and seafood [44]. Caution is therefore needed when interpreting and generalizing results. However, findings regarding macronutrient intake, adherence to recommendations for grains, dairy products and “meat, seafood and eggs” in a random sample of the French population [44] showed estimates equivalent to those in our study.

Another limitation was that the data in our virtual study may not be an accurate reflection of actual label use in real-life settings. For instance, measures of understanding in our epidemiological study probably do not accurately duplicate real-life purchasing situations that involve time pressure and distracting factors forming part of marketing strategies [23], [45], [46]. However, our theoretical approach enables a more rational assessment of label perception than real-life shopping situations in which subjects cannot evaluate the FOP label separately from other packaging features. The issue of accuracy of web-based self-reported data also arises for repeated 24 h dietary records compared to interviews by trained dietitians. However, a pilot study comparing our web-based 24 h record tool with the reference method showed strong agreement between the two methods, while suggesting that the web-based tool may even reduce judgment bias [28]. Moreover, a strength of our study was its reliance on at least three self-reported dietary records provided in the course of one year, which are recommended methods in large epidemiological studies [47] and which enabled a reliable estimation of usual diet [48]. However, it should be noted that under-reporting may still persist and the measure of energy/protein intake may be biased in the absence of objective biomarkers such as doubly labeled water or urinary nitrogen although under-reporters were excluded.

Another strength was that dietary and health data were collected at enrollment in the study, prior to data on understanding and acceptability of FOP labels. Thus, the cohort effect was avoided and participant answers concerning diet and health status were not biased by their participation in the survey on FOP label perception.

It would be useful to carry out equivalent surveys using other types of manufactured products, since soups have a less broad nutrient profile than other products and are generally perceived as healthy, which may have partially biased participant answers. However, the statements we tested resulted in correct answers not directly related to a preconceived “healthy” view of each soup (for instance, the salt content of vegetable soup), thus probably limiting this bias. In addition, among the existing soups on the food market, the different classes of nutritional profiles defined by the SAIN LIM system used in this study were represented, while other alternatives were rare. Indeed, sweet foods and salty foods had low variability in nutrient profiles; a healthy option for many of these products does not exist.

One limitation of our study was that the MTL was not compared to a label format providing more detailed information, such as the Guideline Daily Amount (GDA) [17]. In 2008, at the time of the survey preparation, we chose not to include this label (it was endorsed by the European Union in 2010), as GDA was the least preferred system in the exploratory study conducted among 400 persons for selecting a maximum of five label formats to be tested.

Another weakness of our survey was its within-subject design. This may have induced carryover effects wherein responses to the label format might be influenced by the previous formats seen, especially in terms of understanding. Indeed, the practice effect could lead to increasing the understanding level of participants from one format to the next. However, the latter did not increase with the label rank of presentation, which reasonably let us think that only limited bias would have been found in participant answers. Moreover, the within-subject design was specifically chosen so as to control individual differences in subjects and to reduce error variance associated with them [49].

Consistent with a previous study that evaluated the influence of label formats on energy and nutrient intake [6], our results showed no sizeable difference in nutrient intake and PNNS Guideline Score between perception groups. On the other hand, adherence to specific nutritional recommendations, such as those for fruits and vegetables, dairy products and seafood, significantly varied between clusters. Such findings suggest that perception groups may differ in the way they substitute one food group for another, rather than in terms of overall diet quality.

Overall, interpretation of associations between cluster membership and dietary intake was not clear-cut. Indeed, no perception cluster clearly emerged in terms of dietary pattern, and we observed that each perception group in turn included the highest frequency of individuals who did not meet recommendations for a specific food group. Thus, no specific FOP label seems to be more appropriate than another for reaching populations with a poor diet. However, our findings provide useful information for public health policies, since the implementation of an FOP label depends on priority objectives established to improve food group consumption. For instance, if the public health priority were to increase seafood consumption, then the STL, notably concerning processed ready-cooked dishes, would appear most appropriate. Indeed, this label is more easily accepted by the population that had low consumption of this food group. When we compared them to results before adjustment, differences in diet between clusters after adjustment for socio-demographic and economic factors were significantly reduced, and for some food groups, the relationship changed direction (for instance, the highest percentage of low consumers of fruit and vegetables were found in the ‘favorable to STL’ group before adjustment and in the ‘favorable to CR logo’ group after adjustment). This confirms the strong triangular relationships between socio-demographic and economic profiles, dietary patterns and FOP label perception, and highlights the need to take into account socio-demographic and economic profiles of populations when implementing FOP formats [2], [4], [16], [18], [50]. For instance, a separate analysis showed better acceptability and understanding of STL among young individuals, although CR logo and simple formats (Green Tick and PNNS logo) were best appreciated in elderly [18]. The preference for these FOP labels in elderly may be related to difficulties in processing information in this group or vision problems among very old individuals.

In addition to the fact that MTL is usually best appreciated by the general population [4], [12] our study indicates that the highest percentages of diabetic and hypertensive individuals were found in the ‘favorable to MTL’ group, while the group which favored the STL presented the highest prevalence of low physical activity and obesity, irrespective of socio-demographic and economic characteristics. This suggests that the TL system may have the most effective and eye-catching formats for increasing awareness of healthy choices in groups at high risk of nutrition-related disease. Individuals with diet-related diseases made greater use of labels because the nutritional information was more personally relevant [16], [50], [51]. Indeed, diabetic individuals actively seek out information on sugar content, while hypertensive persons frequently check salt content [52]–[54]. Thus, it can be hypothesized that the MTL label was best appreciated by diabetic and hypertensive individuals due to its nutrient-specific information on the content of packaged foods. This was not observed for hypercholesterolemia, possibly because individuals who declared this disease focused more strongly on overall dietary quality rather than on a specific nutrient for improving their health status.

Only three previous studies explored relationships between types of FOP labels and weight status [6], [20], [24]. Two of them showed no significant association between BMI and label formats in terms of understanding and differentiation of healthiness between products [6], [24]. Results of the third study were in agreement with ours, showing a positive association between a preference for the TL format and BMI [20]. Since the highest prevalence of obesity was found in the ‘favorable to STL’ group, this label, which is simple and easy to use and provides a categorical opinion of the nutritional quality of packaged food, may be the best format for increasing use of nutrition labels and therefore making healthier food choices in this population. The group favorable to STL also had the highest prevalence of low physical activity. Thus, STL seems to be the most appropriate format for reaching this population, at high risk of nutrition-related chronic disease.

Acceptability and understanding of FOP labels depend on individual characteristics. Our findings provide useful information on how to target subgroups at higher risk of diet-related chronic diseases. Compared to persons who favored other FOP labels, individuals who better accepted and understood the TL system were more frequently obese, had lower physical activity or had type-2 diabetes and hypertension. Although no FOP label stood out as being more suitable for reaching populations with poor diet, the acceptability of FOP formats in populations not meeting recommendations differed according to the food group. This provides an indication to public health authorities on how to improve eating habits of specific food groups. However, these results must be confirmed by studies assessing observational use of FOP labels by population groups in real-life settings. Further work exploring the impact of FOP labels upon purchasing behavior across populations from diverse backgrounds and with different health conditions is needed to refine our knowledge when setting up effective nutrition labeling policies.

## Supporting Information

Figure S1 **Example of the three soups tested with Multiple Traffic Lights.** Translation of French words: “Soupe chinoise nouilles, champignons noirs, épices” : Chinese soup noddles, black mushrooms, spices; “Poule au pot aux petits légumes”: boiled chicken with baby vegetables; “Soupe passée aux 9 légumes”: Soup with 9 vegetables; “Sucre ajouté”: Added sugar; “Matières grasses saturées”: Saturated fat; “Sel”: Salt.
(TIF)

Table S1 **Acceptability and understanding of FOP labels by perception cluster, n = 28,952 (Nutrinet-Santé study, 2009–2010).**
(DOCX)

Table S2 **Nutrients intake of perception clusters adjusted for energy intake, n = 28, 952 (Nutrinet-Santé study, 2009–2010).**
(DOCX)

Table S3 **Food groups intakes of perception clusters adjusted for energy intake, n = 28, 952 (Nutrinet-Santé study, 2009–2010).**
(DOCX)

Table S4 **Lifestyle and health profiles of perception clusters, unadjusted, n = 28, 952 (Nutrinet-Santé study, 2009–2010).**
(DOCX)
