# Supplementary figures and images for: Association of Perception of Front-of-Pack Labels with Dietary, Lifestyle and Health Characteristics
Source: PLoS One. 2014 Mar 12;9(3):e90971. doi: 10.1371/journal.pone.0090971 (PMC3951292; doi:10.1371/journal.pone.0090971)

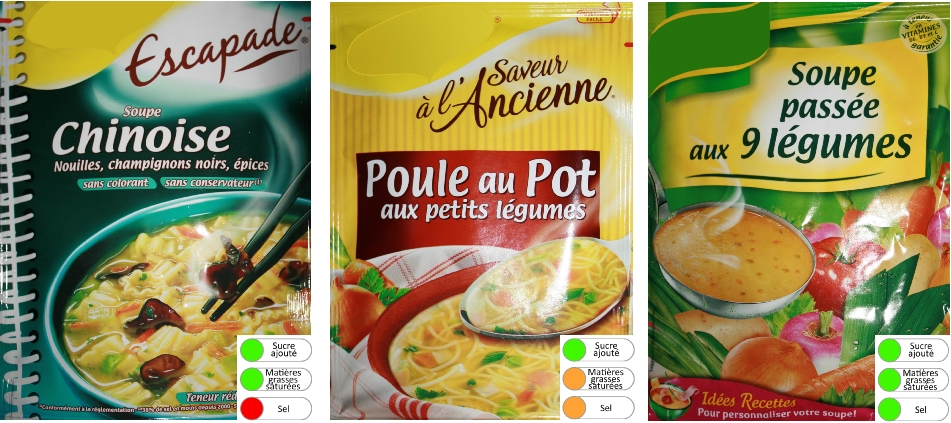

Supplement: Figure S1 — Example of the three soups tested with Multiple Traffic Lights. Translation of French words: “Soupe chinoise nouilles, champignons noirs, épices” : Chinese soup noddles, black mushrooms, spices; “Poule au pot aux petits légumes”: boiled chicken with baby vegetables; “Soupe passée aux 9 légumes”: Soup with 9 vegetables; “Sucre ajouté”: Added sugar; “Matières grasses saturées”: Saturated fat; “Sel”: Salt. (TIF) [file pone.0090971.s001.tif]
