# Supplementary material for: Association of Perception of Front-of-Pack Labels with Dietary, Lifestyle and Health Characteristics
Source: PLoS One. 2014 Mar 12;9(3):e90971. doi: 10.1371/journal.pone.0090971 (PMC3951292; doi:10.1371/journal.pone.0090971)
Supplement: Table S1 — Acceptability and understanding of FOP labels by perception cluster, n = 28,952 (Nutrinet-Santé study, 2009–2010). (DOCX) [file pone.0090971.s002.docx]

Table S1. Acceptability and understanding of FOP labels by perception cluster, n=28 ,952 (Nutrinet-Santé study, 2009-2010)

|  | Total sample  n=28 952 (%) | "Favorable to MTL" group ^a^  n=19 842 (%) | | "Favorable to green tick and PNNS logo" group^b^  n=5 932 (%) | "Favorable to STL" group^c^  n=2 973 (%) | "Favorable to CR logo" group^d^  n=808 (%) |
| --- | --- | --- | --- | --- | --- | --- |
| *I prefer this label* | | | | | | |
| MTL | 59.40 | 87.04 | | 9.93 | 3.84 | 13.01 |
| Green Tick | 13.60 | 5.18 | | 45.56 | 2.49 | 4.56 |
| PNNS logo | 8.28 | 2.50 | | 30.55 | 0.36 | 1.20 |
| STL | 12.44 | 3.48 | | 4.11 | 93.09 | 3.88 |
| CR logo | 3.43 | 0.46 | | 0.92 | 0.16 | 76.16 |
| None | 2.84 | 1.34 | | 8.94 | 0.06 | 1.19 |
| *I would like to see this label on the front of packages* | | | | |  |  |
| MTL | 58.67 | 86.36 | | 8.15 | 6.93 | 7.76 |
| Green Tick | 10.64 | 2.48 | | 40.62 | 1.33 | 3.76 |
| PNNS logo | 7.75 | 2.33 | | 28.52 | 1.42 | 2.96 |
| STL | 10.41 | 2.08 | | 1.59 | 85.85 | 3.23 |
| CR logo | 2.91 | 0.11 | | 0.17 | 0.09 | 72.90 |
| None | 9.61 | 6.64 | | 20.95 | 4.38 | 9.35 |
| *This label is helping me to choose healthier products* | | | | | |  |
| MTL | 59.35 | 86.23 | | 10.40 | 8.37 | 11.13 |
| Green Tick | 11.49 | 3.92 | | 39.70 | 2.30 | 4.24 |
| PNNS logo | 6.85 | 1.48 | | 26.62 | 0.54 | 2.58 |
| STL | 11.25 | 3.12 | | 3.21 | 86.21 | 1.61 |
| CR logo | 3.09 | 0.24 | | 0.51 | 0.25 | 73.01 |
| None | 7.98 | 5.01 | | 19.56 | 2.34 | 7.42 |
| *I don’t like this label* | | | | | | |
| MTL | 4.55 | 0.63 | | 12.97 | 7.67 | 15.29 |
| Green Tick | 5.57 | 6.52 | | 2.67 | 5.28 | 14.51 |
| PNNS logo | 21.04 | 22.78 | | 10.44 | 21.75 | 49.50 |
| STL | 7.10 | 7.62 | | 7.70 | 0.06 | 12.97 |
| CR logo | 58.53 | 61.00 | | 58.92 | 63.59 | 1.74 |
| None | 2.91 | 1.45 | | 7.30 | 1.66 | 5.98 |
| **Attractiveness** | | | | | | |
| *I can rely on this label* | | | | | | |
| MTL | 44.38 | 64.74 | | 5.53 | 8.67 | 10.43 |
| Green Tick | 16.19 | 9.55 | | 39.80 | 10.51 | 10.10 |
| PNNS logo | 20.63 | 17.01 | | 36.24 | 13.48 | 12.23 |
| STL | 8.74 | 2.61 | | 2.60 | 64.71 | 3.44 |
| CR logo | 2.71 | 0.46 | | 0.84 | 0.18 | 57.70 |
| None | 7.35 | 5.62 | | 14.99 | 2.44 | 6.10 |
| *This label provides all the information I need* | | | | | |  |
| MTL | 65.07 | 88.30 | | 21.99 | 23.84 | 20.75 |
| Green Tick | 7.44 | 1.53 | | 29.13 | 0.69 | 2.51 |
| PNNS logo | 4.77 | 0.74 | | 19.52 | 0.45 | 0.91 |
| STL | 8.86 | 1.51 | | 4.22 | 69.91 | 2.43 |
| CR logo | 2.79 | 0.39 | | 0.73 | 0.74 | 60.31 |
| None | 11.07 | 7.53 | | 24.41 | 4.36 | 13.10 |
| **Perceived cognitive workload** | | | | | | |
| *This label is hard to understand* | | | | | | |
| MTL | 7.05 | 0.65 | | 18.77 | 17.31 | 23.02 |
| Green Tick | 1.36 | 1.88 | | 0.37 | 0.13 | 1.20 |
| PNNS logo | 4.89 | 4.36 | | 4.58 | 4.72 | 16.05 |
| STL | 0.84 | 1.19 | | 0.09 | 0.00 | 1.45 |
| CR logo | 55.72 | 61.44 | | 45.87 | 60.44 | 2.33 |
| None | 30.14 | 30.48 | | 30.33 | 17.41 | 55.94 |
| *This label requires a lot of time for understanding* | | | | | | |
| MTL | 12.52 | 2.04 | 31.09 | | 31.99 | 35.23 |
| Green Tick | 1.72 | 2.13 | 0.83 | | 0.36 | 3.22 |
| PNNS logo | 4.68 | 4.23 | 4.27 | | 5.75 | 11.83 |
| STL | 1.31 | 1.77 | 0.36 | | 0.18 | 1.84 |
| CR logo | 50.83 | 59.06 | 36.88 | | 45.66 | 3.57 |
| None | 28.93 | 30.77 | 26.57 | | 16.06 | 44.31 |
| *This label makes me feel uncomfortable* | | | | | | |
| MTL | 5.58 | 2.35 | 11.61 | | 11.49 | 11.04 |
| Green Tick | 1.49 | 1.58 | 1.04 | | 2.04 | 0.96 |
| PNNS logo | 1.99 | 1.83 | 1.68 | | 3.63 | 2.22 |
| STL | 13.08 | 14.72 | 12.33 | | 4.00 | 13.03 |
| CR logo | 3.72 | 3.38 | 4.73 | | 4.52 | 1.56 |
| None | 74.15 | 76.14 | 68.61 | | 74.32 | 71.19 |
| **Objective understanding** | | | | | | |
| *High level of understanding (4-5 points)* | | | | | | |
| MTL | 55.06 | 64.25 | 34.40 | | 46.34 | 38.36 |
| Green Tick | 64.48 | 67.50 | 56.79 | | 65.33 | 54.51 |
| PNNS logo | 61.87 | 67.03 | 48.62 | | 60.14 | 53.71 |
| STL | 60.60 | 67.98 | 41.11 | | 61.02 | 44.38 |
| CR logo | 51.90 | 57.63 | 36.31 | | 50.23 | 47.01 |
| *Low level of understanding (0-2 points)* | | | | | | |
| MTL | 21.25 | 11.64 | 42.57 | | 30.04 | 41.07 |
| Green Tick | 11.36 | 9.53 | 15.55 | | 12.64 | 15.48 |
| PNNS logo | 11.63 | 9.28 | 17.99 | | 11.33 | 16.18 |
| STL | 18.65 | 13.06 | 32.66 | | 19.85 | 31.18 |
| CR logo | 28.37 | 23.41 | 31.74 | | 11.30 | 25.96 |

a MTL, multiple traffic lights

b PNNS, French Nutrition and Health Program

c STL, simple traffic lights

d CR, color range
