# Supplementary material for: Association of Perception of Front-of-Pack Labels with Dietary, Lifestyle and Health Characteristics
Source: PLoS One. 2014 Mar 12;9(3):e90971. doi: 10.1371/journal.pone.0090971 (PMC3951292; doi:10.1371/journal.pone.0090971)
Supplement: Table S2 — Nutrients intake of perception clusters adjusted for energy intake, n = 28, 952 (Nutrinet-Santé study, 2009–2010). (DOCX) [file pone.0090971.s003.docx]

Table S2. Nutrients intake of perception clusters, adjusted for energy intake, adjusted for energy intake, n=28, 952 (Nutrinet-Santé study, 2009-2010)

|  | Total sample   n=28 952 | | "Favorable to MTL" group ^a^  n=19 842 | | "Favorable to green tick and PNNS logo" group ^b^  n=5 932 | | "Favorable to STL" group ^c^  n=2 973 | | "Favorable to CR logo" group ^d^  n=808 | |  |
| --- | --- | --- | --- | --- | --- | --- | --- | --- | --- | --- | --- |
|  | Mean | SE | Mean | SE | Mean | SE | Mean | SE | Mean | SE | P-value |
|  |  |  |  |  |  |  |  |  |  |  |  |
| **Energy intake**^e^ | 2087.22 | 426.75 | 2095.06 | 3.85 | 2045.64 | 6.72 | 2071.57 | 9.90 | 2176.59 | 16.04 | <0.0001 |
|  |  |  |  |  |  |  |  |  |  |  |  |
| **Proteins (% of energy intake)** ^e^ | 16.64 | 0.06 | 16.62 | 0.02 | 16.66 | 0.04 | 16.51 | 0.06 | 16.76 | 0.11 | 0.07 |
|  |  |  |  |  |  |  |  |  |  |  |  |
| **Lipids (% of energy intake)** ^e^ | 37.61 | 0.11 | 37.84 | 0.04 | 37.42 | 0.08 | 38.20 | 0.11 | 36.98 | 0.20 | <0.0001 |
|  |  |  |  |  |  |  |  |  |  |  |  |
| **Saturated fat (% of energy intake)** ^e^ | 15.08 | 0.06 | 15.13 | 0.02 | 14.84 | 0.04 | 15.41 | 0.06 | 14.94 | 0.10 | <0.0001 |
|  |  |  |  |  |  |  |  |  |  |  |  |
| **Carbohydrates (% of energy intake)** ^e^ | 42.06 | 0.12 | 42.08 | 0.05 | 41.94 | 0.09 | 42.16 | 0.12 | 42.06 | 0.23 | 0.46 |
|  |  |  |  |  |  |  |  |  |  |  |  |
| **Simple carbohydrates (% of energy intake)** ^e^ | 19.35 | 0.09 | 19.57 | 0.04 | 19.29 | 0.07 | 19.90 | 0.10 | 18.64 | 0.16 | <0.0001 |
|  |  |  |  |  |  |  |  |  |  |  |  |
| **Fibers (g/day)** ^f^ | 19.71 | 0.12 | 19.55 | 0.05 | 20.34 | 0.08 | 18.74 | 0.13 | 20.21 | 0.20 | <0.0001 |
|  |  |  |  |  |  |  |  |  |  |  |  |
| **Sodium (mg/day)** ^f^ | 2103.31 | 39.47 | 2170.92 | 15.19 | 2135.18 | 28.72 | 2033.62 | 38.34 | 2073.51 | 75.65 | 0.54 |
|  |  |  |  |  |  |  |  |  |  |  |  |
| **Cholesterol (mg/day)** ^f^ | 494.48 | 3.19 | 498.24 | 1.23 | 495.80 | 2.32 | 495.50 | 3.10 | 488.38 | 6.11 | 0.36 |
|  |  |  |  |  |  |  |  |  |  |  |  |
| **Iron (mg/day)** ^f^ | 12.82 | 0.07 | 12.82 | 0.03 | 12.92 | 0.05 | 12.43 | 0.07 | 13.10 | 0.12 | <.0001 |
|  |  |  |  |  |  |  |  |  |  |  |  |
| **Calcium (mg/day)** ^f^ | 905.84 | 4.53 | 905.28 | 1.97 | 902.51 | 3.32 | 900.45 | 4.89 | 915.12 | 7.93 | 0.39 |
|  |  |  |  |  |  |  |  |  |  |  |  |

a MTL, multiple traffic lights

b PNNS, French Nutrition and Health Program

c STL, simple traffic lights

d CR, color range

e Unadjusted model

f The model was adjusted for energy intake
