# Supplementary material for: Association of Perception of Front-of-Pack Labels with Dietary, Lifestyle and Health Characteristics
Source: PLoS One. 2014 Mar 12;9(3):e90971. doi: 10.1371/journal.pone.0090971 (PMC3951292; doi:10.1371/journal.pone.0090971)
Supplement: Table S3 — Food groups intakes of perception clusters adjusted for energy intake, n = 28, 952 (Nutrinet-Santé study, 2009–2010). (DOCX) [file pone.0090971.s004.docx]

Table S3. Food groups intakes of perception clusters, adjusted for energy intake, n=28, 952 (Nutrinet-Santé study, 2009-2010)a

|  | Total sample  n=28 952 (%^f^ ) |  | "Favorable to MTL" group^b^  n=19 842 (%^f^ ) | "Favorable to green tick and PNNS logo" group ^c^  n=5 932 (%^f^ ) | "Favorable to STL" group ^d^  n=2 973 (%^f^ ) | "Favorable to CR logo" group ^e^  n=808 (%^f^ ) |
| --- | --- | --- | --- | --- | --- | --- |
|  |  |  |  |  |  |  |
| **Fruits and vegetables (servings/d)** |  |  |  |  |  |  |
| Low consumption (≥0-<3.5 ) | 27.03 |  | 26.16 | 24.85 | 30.40 | 26.72 |
| Moderate consumption (≥3.5-<5) | 20.58 |  | 23.19 | 21.71 | 22.74 | 14.65 |
| Adherence to the recommendation (≥ 5) | 52.39 |  | 50.65 | 53.44 | 46.86 | 58.63 |
|  |  |  |  |  |  |  |
| **Grains (servings/d)** |  |  |  |  |  |  |
| Low consumption (≥0-<3 ) | 52.28 |  | 54.09 | 49.82 | 57.18 | 48.03 |
| Adherence to the recommendation (≥3-<6) | 44.00 |  | 42.57 | 45.94 | 41.67 | 45.82 |
| High consumption (≥ 6) | 3.72 |  | 3.34 | 4.24 | 1.15 | 6.15 |
|  |  |  |  |  |  |  |
| **Milk and dairy products (servings/d)** |  |  |  |  |  |  |
| Low consumption (≥0-2.5> ) | 53.00 |  | 51.79 | 52.76 | 53.65 | 53.79 |
| Adherence to the recommendation (<55y: ≥2.5-<3.5; ≥55 y: ≥2.5-<4.5) | 30.71 |  | 29.86 | 33.71 | 28.35 | 30.92 |
| High consumption (<55y: > 3.5; ≥55 y: >4.5) | 16.29 |  | 18.35 | 13.53 | 18.00 | 15.29 |
|  |  |  |  |  |  |  |
| **Meat and poultry. seafood. and eggs (servings/d)** |  |  |  |  |  |  |
| Low consumption (≥0-1> ) | 24.78 |  | 26.82 | 24.06 | 25.19 | 23.07 |
| Adherence to the recommendation (≥1-<2) | 59.34 |  | 57.75 | 59.20 | 60.37 | 60.04 |
| High consumption (>2) | 15.88 |  | 15.43 | 16.74 | 14.44 | 16.89 |
|  |  |  |  |  |  |  |
| **Seafood (servings/wk)** |  |  |  |  |  |  |
| Low consumption (<2) | 50.25 |  | 50.26 | 46.37 | 59.57 | 44.81 |
| Adherence to the recommendation (≥ 2) | 49.75 |  | 49.74 | 53.63 | 40.43 | 55.19 |
|  |  |  |  |  |  |  |
| **Alcohol (Ethanol g/d)** |  |  |  |  |  |  |
| High consumption (>20 for women and >30 for men) | 12.09 |  | 11.15 | 14.43 | 10.78 | 12.01 |
| Moderate consumption (≤ 20 for women and ≤30 for men) | 71.39 |  | 71.59 | 70.54 | 68.94 | 74.5 |
| Abstainers and irregular consumers (< once a week) | 16.52 |  | 17.26 | 15.03 | 20.28 | 13.49 |
|  |  |  |  |  |  |  |

a All p-values were < 0.0001

b MTL, multiple traffic lights

c PNNS, French Nutrition and Health Program

d STL, simple traffic lights

e CR, color range

f For each variable, percentages were adjusted for energy intake
