# Supplementary material for: Association of Perception of Front-of-Pack Labels with Dietary, Lifestyle and Health Characteristics
Source: PLoS One. 2014 Mar 12;9(3):e90971. doi: 10.1371/journal.pone.0090971 (PMC3951292; doi:10.1371/journal.pone.0090971)
Supplement: Table S4 — Lifestyle and health profiles of perception clusters, unadjusted, n = 28, 952 (Nutrinet-Santé study, 2009–2010). (DOCX) [file pone.0090971.s005.docx]

Table S4. Lifestyle and health profiles of perception clusters, unadjusted, n=28, 952 (Nutrinet-Santé study, 2009-2010)^a^

|  | Total sample  n=28 952 (%^f^ ) | |  | "Favorable to MTL" group^b^  n=19 842 (%^f^ ) | "Favorable to green tick and PNNS logo" group ^c^  n=5 932 (%^f^ ) | "Favorable to STL" group ^d^  n=2 973 (%^f^ ) | "Favorable to CR logo" group ^e^  n=808 (%^f^ ) |
| --- | --- | --- | --- | --- | --- | --- | --- |
|  |  |  |  |  |  |  |  |
|  |  | |  |  |  |  |  |
| **Physical activity level (min/d)** | |  |  |  |  |  |  |
| Low (<30) | 16.96 | |  | 17.70 | 15.33 | 20.36 | 14.45 |
| Moderate (≥30-<60) | 16.14 | |  | 18.77 | 15.36 | 17.81 | 12.62 |
| high (≥ 60) | 66.9 | |  | 63.53 | 69.32 | 61.83 | 72.93 |
|  |  | |  |  |  |  |  |
| **Body mass status (kg/m2)** | |  |  |  |  |  |  |
| Normal (< 25) | 65.2 | |  | 67.51 | 63.88 | 69.13 | 60.27 |
| Overweight (≥25-<30) | 25.85 | |  | 24.13 | 27.19 | 20.97 | 31.12 |
| Obese (≥ 30) | 8.95 | |  | 8.37 | 8.94 | 9.91 | 8.61 |
|  |  | |  |  |  |  |  |
| **Self-reported type 2 diabetes** | 2.00 | |  | 2.20 | 2.07 | 1.48 | 2.24 |
|  |  | |  |  |  |  |  |
| **Self-reported hypertension** | 12.01 | |  | 10.19 | 12.96 | 8.62 | 16.26 |
|  |  | |  |  |  |  |  |
| **Self-reported hypercholesterolemia** | 13.09 | |  | 11.48 | 14.91 | 8.57 | 17.38 |
|  |  | |  |  |  |  |  |

a All p-values were < 0.0001

b MTL, multiple traffic lights

c PNNS, French Nutrition and Health Program

d STL, simple traffic lights

e CR, color range

f For each variable, percentages were unadjusted
